# Determination of Effect of *Morus alba* Extract and *Lactobacillus rhamnosus* on Vaginal Microbiota and SAP Gene (1–10) Profile With *Candida albicans* Exosome Administration in Rats

**DOI:** 10.1002/mbo3.70120

**Published:** 2025-11-21

**Authors:** Mahmut Ucar, Demet Celebi, Ozgur Celebi, Sumeyye Baser, Mustafa Can Guler, Ayhan Tanyeli, Metin Kılıclıoglu, Ahmet Yılmaz, Serkan Yıldırım

**Affiliations:** ^1^ Department of Medical Microbiology Atatürk University, Faculty of Medicine Erzurum Turkey; ^2^ Department of Microbiology Atatürk University, Faculty of Veterinary Erzurum Turkey; ^3^ Department of Pharmaceutical Microbiology Erzincan Binali Yıldırım University, Faculty of Pharmacy Erzincan Turkey; ^4^ Department of Physiology Atatürk University, Faculty of Medicine Erzurum Turkey; ^5^ Department of Pathology Atatürk University, Faculty of Veterinary Erzurum Turkey; ^6^ Vocational School of Health Services Atatürk University Erzurum Turkey

**Keywords:** *Candida albicans*, exosomes, *Lactobacillus rhamnosus*, *Morus alba*, *SAP* genes, vaginal microbiota

## Abstract

Vaginal microbiota is essential for mucosal immunity, pathogen defense, and homeostasis. Disruption of this balance can promote opportunistic infections, notably by *Candida albicans*. This study investigates the therapeutic potential of *Morus alba* (MA) extract and *Lactobacillus rhamnosus* (LR) in a rat model exposed to *Candida albicans* exosomes (CAE). CAE induced a 1.3–5.1‐fold upregulation in SAP1–10 gene expression, with SAP4 showing the highest increase (*p* ≤ 0.05). MA and LR monotherapies selectively suppressed SAP6 (41%) and SAP4 (23%), respectively. Notably, combination therapy (CAE+MA+LR) synergistically inhibited all SAP genes (0.8–1.1‐fold, *p* ≤ 0.05). 16S rRNA analysis showed that LR‐containing groups maintained Lactobacillales abundance (37.77%–38.7%), while MA reduced Mycobacteriales by 68.5% (*p* = 0.004). Microbial diversity was lower in the MA group (*H* = 3.242) but higher in LR groups (H = 5.493–5.598). Histopathology revealed severe ovarian inflammation in the CAE group (87.4%, *p* = 0.0022) with a 4.2‐fold increase in IL‐6, while combination therapy reduced inflammation and IL‐6 levels by 60%–72% (*p* ≤ 0.05). Immunofluorescence confirmed downregulation of TLR4 and Caspase‐3 with treatment. FIB‐SEM and NTA analyses showed that CAE exosomes had a heterogeneous morphology (99.3 ± 31.1 nm) and high polydispersity (PDI = 0.31). These results suggest that MA and LR synergistically reduce fungal virulence, restore microbial balance, and offer a promising adjuvant strategy against vaginal candidiasis.

## Graphical abstract

The vaginal microbiota plays a crucial role in mucosal immunity and defense against pathogens. Disruption of this balance by *Candida albicans* exosomes (CAE) upregulates virulence‐related SAP1–10 genes and induces inflammation. In a rat model, treatment with *Morus alba* (MA) extract and *Lactobacillus rhamnosus* (LR) demonstrated selective suppression of SAP gene expression and restoration of microbial homeostasis. Combination therapy (MA + LR) synergistically reduced SAP1–10 expression, IL‐6, TLR4, and Caspase‐3 levels, leading to decreased ovarian inflammation and reestablished Lactobacillales abundance. These results highlight the potential of MA and LR as a synergistic adjuvant approach against CAE‐induced vaginal dysbiosis.

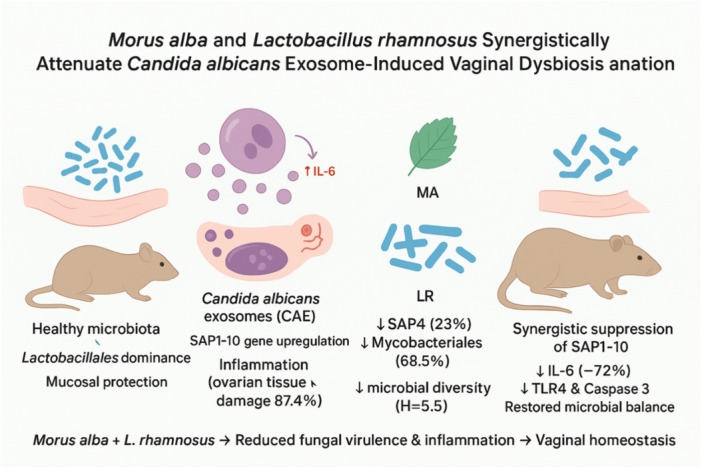

## 1 Introduction

Vaginal microbiota plays a fundamental role in maintaining the health and stability of the female reproductive tract. Acting as a protective barrier, it regulates mucosal immunity, prevents pathogen colonization, and contributes significantly to gynecological health and overall well‐being (Cocomazzi et al. 2023). However, disturbances in this delicate microbial ecosystem can lead to dysbiosis, creating an environment that favors the overgrowth of opportunistic pathogens, ultimately resulting in infections (Tuniyazi and Zhang 2023). Modulation of the vaginal microbiota through probiotic therapy has emerged as a promising approach to restoring microbial balance and influencing humoral immune responses (Mykhailyshyn et al. 2023). The ability to accurately identify microbial shifts within the vaginal ecosystem is crucial for understanding the transition from beneficial to pathogenic microorganisms, which is key to preventing infections (Javadi et al. 2024). Thus, an in‐depth understanding of vaginal microbiota dynamics and its interaction with pathogens is essential for developing novel therapeutic interventions.

*Morus alba* (white mulberry) has been widely used in traditional medicine due to its extensive pharmacological properties, including anti‐inflammatory, antioxidant, and antimicrobial effects (Kwon et al. 2022). Emerging evidence suggests that *Morus alba* extracts can influence the composition and function of the microbiota (Du et al. 2022), with studies highlighting their modulatory effects on gut microbiota and metabolic processes, particularly in conditions such as diabetes (Zheng et al. 2023) and obesity (Wan et al. 2022). In one study, diabetic rats treated with *Morus alba* leaf extract at a dose of 600 mg/kg showed significant decreases in blood glucose, HbA1c, triglyceride and LDL levels. In addition, the diameter of the islets of Langerhans and the number of β‐cells approached control levels (Mohammadi and Naik 2008). In another study, *Morus alba* stem extract reduced nitric oxide production by suppressing iNOS and COX‐2 expression in LPS‐stimulated RAW 264.7 macrophage cells. This indicates the anti‐inflammatory potential of the extract (Soonthornsit et al. 2017). In the analysis of leaf, stem and fruit extracts of *Morus alba*, especially ethanolic extracts exhibited potent antioxidant and moderate antimicrobial activities due to their high phenolic and flavonoid contents (Wang et al. 2012). In the STAM mouse model, hepatocellular carcinoma development was prevented in mice fed 1% *Morus alba* leaf powder, and only fat accumulation and adenoma formation were observed. This suggests that the extract may prevent the progression of liver diseases (Soonthornsit et al. 2017).

A vaginal microbiota dominated by *Lactobacillus* species is closely linked to vaginal health, as these beneficial bacteria inhibit the growth of pathogens like *Candida albicans* (Takano et al. 2023). The presence of *Lactobacillus* species is essential for maintaining a stable and acidic vaginal environment, thereby preventing microbial infections (Chee et al. 2020). Among these, *Lactobacillus rhamnosus* is a well‐characterized probiotic strain known for its beneficial effects on both gut and vaginal health. It contributes to the maintenance of vaginal pH, pathogen inhibition, and immune system modulation (Puebla‐Barragan et al. 2021). The administration of *L. rhamnosus* has demonstrated promising potential in preventing and treating infections by restoring vaginal microbiota homeostasis (S. Yang et al. 2020). Additionally, its antifungal activity has been found to be effective in combating *Candida albicans*‐associated infections (Rose Jørgensen et al. 2020), suppressing fungal overgrowth, protecting against oral infections, and restoring microbial equilibrium (Lactobacillus Rhamnosus GG Role in the Suppression of *Candida albicans* Causing Candidiasis Thrush 2021). The significance of *L. rhamnosus* in probiotic research has been emphasized by its widespread isolation from various sources, including the human gastrointestinal tract and dairy products (Chung et al. 2023). In one study, *L. rhamnosus* was effective in preventing dental caries by reducing biofilm formation and decreasing lactic acid production. LGG strain reduced enamel mineral loss and decreased lesion depth in a rat model (Chen et al. 2024). In another study, in chicks infected with *Salmonella typhimurium*, *L. rhamnosus* administration reduced diarrhea, increased weight gain and reduced inflammation in the intestinal mucosa. It also strengthened gut barrier integrity and increased microbiota diversity (Peng et al. 2022). In a study on porcine intestinal epithelial cells, *L. rhamnosus* reduced Salmonella and *E. coli*‐induced inflammation, decreased ROS production and modulated cytokine responses (G.‐Y. Yang et al. 2017). This demonstrates the gut health‐promoting effects of probiotics (Palkovicsné Pézsa et al. 2023; G.‐Y. Yang et al. 2017). In a study in ovariectomized rats, *L. rhamnosus* GG administration reduced bone loss by regulating Th17/Treg cell balance and modulating gut microbiota (Guo et al. 2023). These scientific findings demonstrate the diverse biological effects of *Morus alba* extract and *L. rhamnosus* and scientifically support the testing of these ingredients in animal models.

Exosomes, a subset of extracellular vesicles (EVs) measuring 30–150 nm, serve as key mediators of intercellular communication by transporting proteins, lipids, and RNA. These vesicles influence various physiological and pathological processes, including immune responses. *Candida albicans* secretes EVs that differ based on its morphological state (i.e., yeast vs. hyphal forms). Hyphal EVs (HEVs) tend to be smaller and more diverse in protein content, contributing to increased fungal virulence, whereas yeast EVs (YEVs) are larger and primarily enriched with cell wall proteins. HEVs are of particular interest due to their cytoplasmic protein content and active 20S proteasome complex, which may play a role in immune evasion (Martínez‐López et al. 2022).

A major virulence factor of *Candida albicans* is the secreted aspartyl proteinase (SAP) family, which plays a critical role in tissue invasion and immune evasion. The expression of *SAP* genes is tightly regulated and significantly influences infection severity and host immune response (Ahmed et al. 2024; Safiya S et al. 2023). SAPs are involved in host adhesion, tissue degradation, and biofilm formation, making them key targets for antifungal therapies (Fathy et al. 2023). Consequently, inhibiting *SAP* gene expression has been explored as a potential therapeutic strategy against *Candida* infections (Gholam 2022; Hartanto et al. 2022).

This study aims to investigate the effects of *Morus alba* extract and *Lactobacillus rhamnosus* on vaginal microbiota composition and *SAP* gene expression (1–10) in a *Candida albicans* exosome (CAE)‐induced rat model. By elucidating these interactions, we seek to identify potential synergistic therapeutic strategies that promote vaginal health and offer novel approaches for preventing and managing fungal infections. This study will provide new insights into the interplay between probiotics, phytotherapeutic agents, and fungal virulence factors, paving the way for alternative interventions in vaginal dysbiosis.

## 2 Results

### 2.1 Focused Ion Beam—Scanning Electron Microscopy (FIB‐SEM) Results

**Figure 1:**
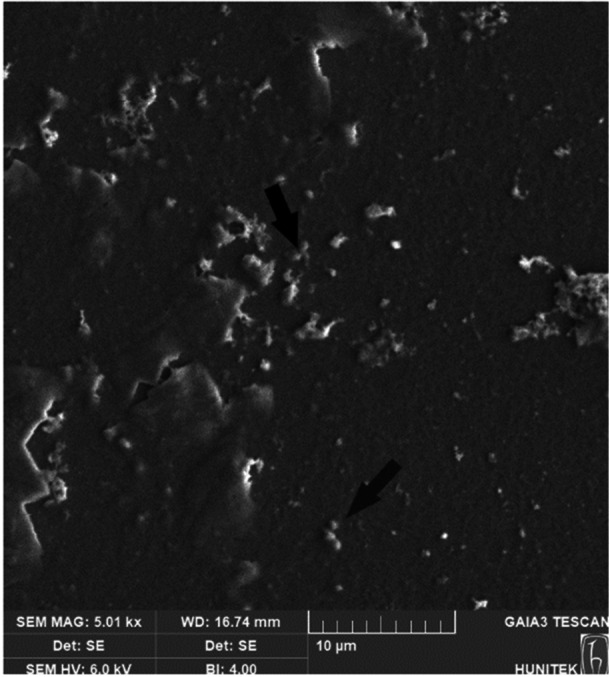
SEM image of *Candida albicans* exosomes.

The SEM image of exosomes isolated from *Candida albicans* is presented in Figure 1. The FIB‐SEM (Focused Ion Beam—SEM) analysis clearly visualizes the spherical and amorphous structures of the exosomes, confirming their nanoparticle nature. These results validate the successful isolation of *Candida albicans*‐derived exosomes and provide insight into their morphological characteristics.

### 2.2 Nanoparticle Tracking Analysis (NTA) Results

**Figure 2:**
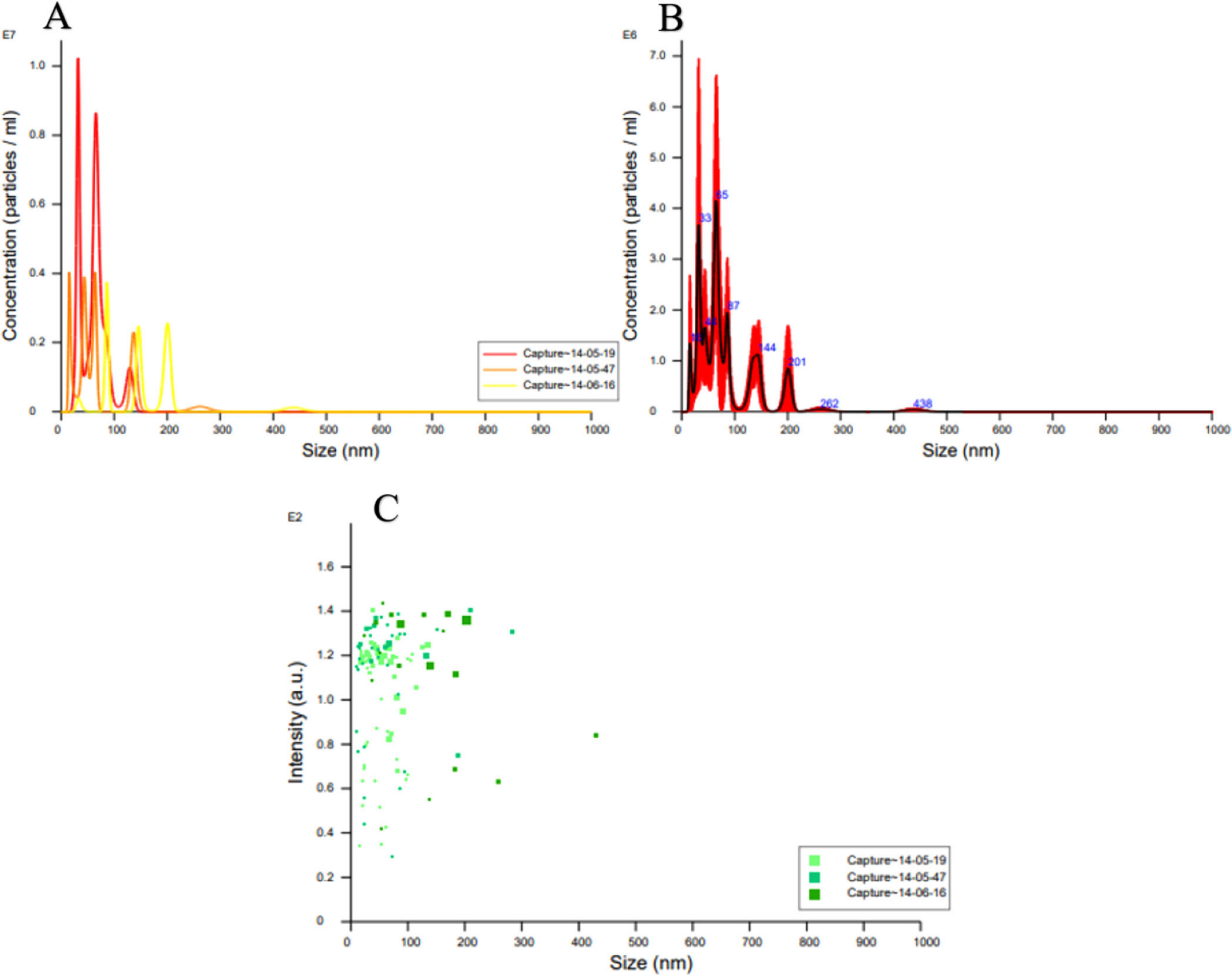
NTA analysis results.

NTA revealed that CAEs exhibit a heterogeneous size distribution (Figure 2). The key parameters obtained from NTA analysis include:

- Mean Diameter: 99.3 ± 31.1 nm (*Raw data: 85.6 nm*);
- Mode Diameter: 60.9 ± 15.7 nm (*Raw data: 64.9 nm*);
- Standard Deviation: 55.6 ± 16.0 nm, indicating high particle polydispersity.

The size distribution analysis indicated that:

- 10% of exosomes had a diameter of < 44.1 nm;
- 50% of exosomes had a diameter of < 90.2 nm;
- 90% of exosomes had a diameter of < 146.7 nm.

Additionally, the exosome concentration and particle density were determined as follows:

- Particle Density: 1.82 ± 5.41 × 10⁸ particles/mL;
- Particles per Square: 2.5 ± 0.6 particles/square;
- Focus Centers: 6.5 ± 2.2 centers/square.

These results demonstrate that CAEs exhibit a broad size range and high polydispersity, consistent with the heterogeneity observed in EV populations.

### 2.3 Real‐Time PCR Results

**Figure 3:**
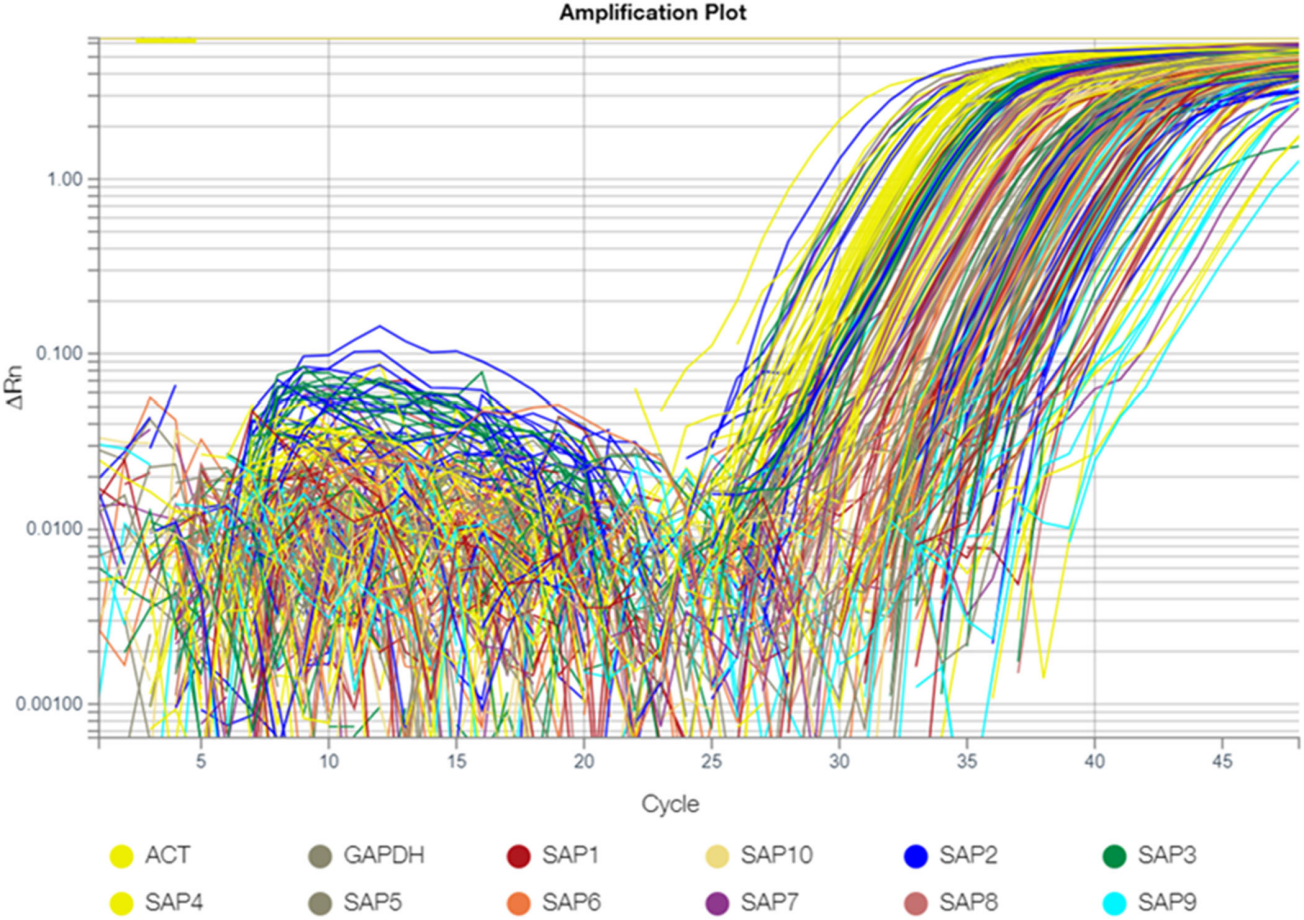
Amplification data and ct values of *SAP1–10* endogenous control actin and GAPDH gene expressions in blood samples of control and treatment groups.

The expression levels of *SAP1–10* genes in the control and treatment groups were analyzed using quantitative real‐time PCR (qRT‐PCR). The amplification curve obtained from qRT‐PCR is presented in Figure 3.

**Figure 4:**
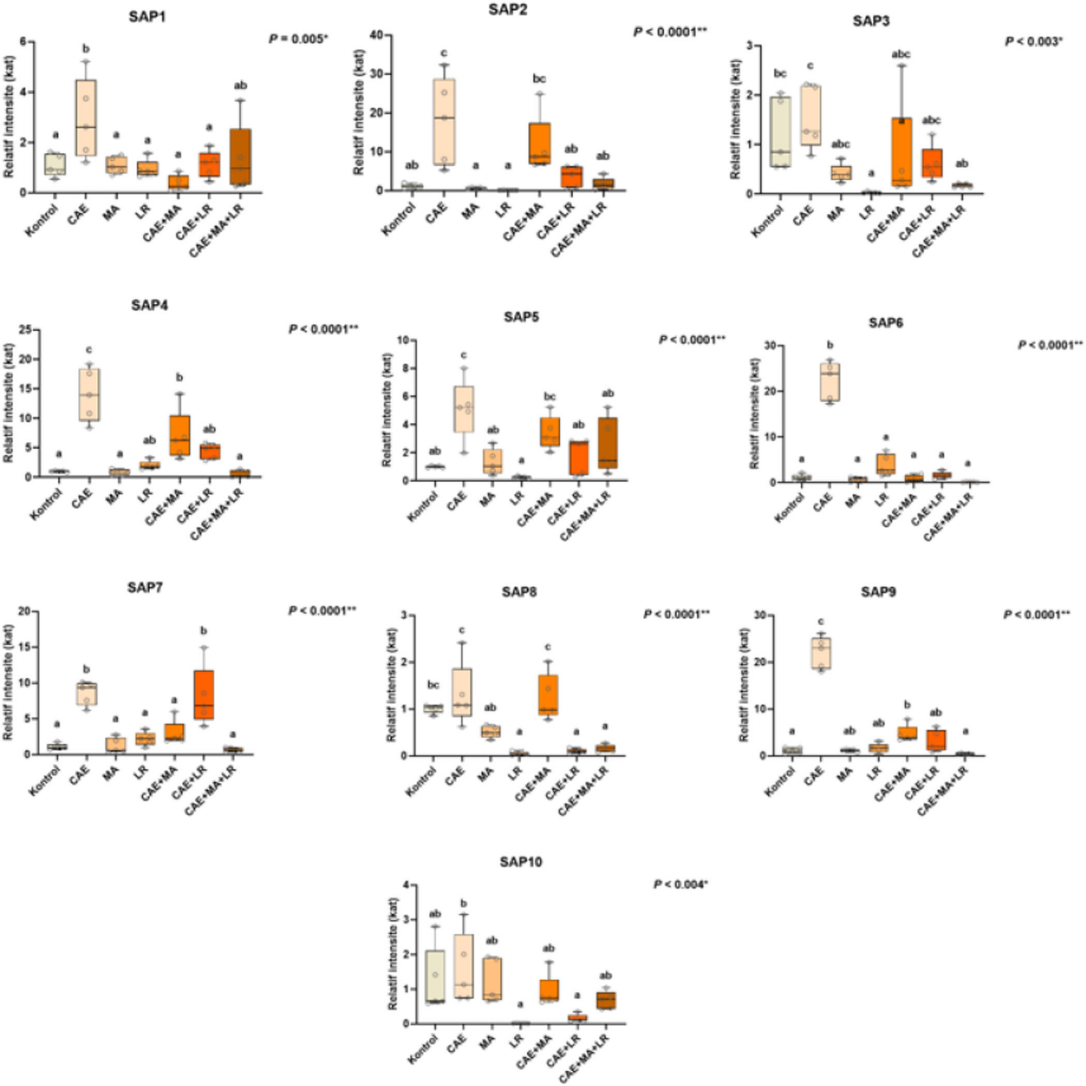
Relative fold increase/decrease values of *SAP 1–10* gene expressions determined in the blood tissues of control, *Candida albicans* Exosome (CAE), *Morus alba* extract (MA), *Lactobacillus rhamnosus* (LR), CAE+MA, CAE+LR, CAE+LR, CAE+MA+LR treated rats (data were normalized with actin and GPH mRNA levels by multiple control method, data mean±SD), * means indicated with different letters are statistically different, one‐way ANOVA, Duncan's test, *p* ≤ 0.05.

The relative expression dynamics of the *SAP1‐10* gene family in blood tissue from rats treated with *Candida albicans* exosomes (CAEs) and therapeutic agents are shown in Figure 4. Gene expression data were normalized to actin and GAPDH mRNA levels. A statistically significant increase (*p* ≤ 0.05) in *SAP* gene expression was observed following CAE administration, with SAP4 exhibiting the highest upregulation (5.1‐fold increase).

Gene Expression Modulation by Treatment Groups:

*Morus alba* (MA) extract Group:

- Exhibited the highest suppression of *SAP6* (1.7‐fold, *p* ≤ 0.05).
- Showed limited effects on *SAP1‐3* (2.9–3.1‐fold inhibition).
- The suppression of *SAP5* (1.4‐fold) was linked to MA's polyphenolic content (e.g., quercetin, morin), which is known to inhibit fungal protease activity and interfere with cell wall synthesis.

*Lactobacillus rhamnosus* (LR) Group:

- Specifically inhibited *SAP4* expression (1.3‐fold, *p* ≤ 0.05).
- Showed significant suppression of *SAP4*, *SAP7*, and *SAP8* (1.3–1.6 fold decrease).
- The suppression of *SAP* genes in the CAE+LR group was attributed to LR's immunomodulatory effects, balancing Th1/Th2 immune responses and mitigating *Candida albicans*‐driven inflammation.

CAE+MA Group:

- Showed suppression of *SAP1*, *SAP5*, and *SAP9* in the range of 1.2–1.5‐fold.
- This effect is consistent with MA polyphenols interfering with fungal cell wall biosynthesis and protease inhibition mechanisms.

CAE+LR Group:

- Reduced *SAP4*, *SAP7*, and *SAP8* gene expression by 1.3–1.6‐fold.
- The most pronounced suppression was observed in *SAP4* (1.3‐fold inhibition), indicating a direct link between *SAP4* and immune activation.

CAE+MA+LR (Combination Therapy) Group:

- Demonstrated comprehensive SAP gene suppression (0.8–1.1 fold, *p* ≤ 0.05) across all *SAP1‐10* genes.
- The decrease in *SAP10* expression (0.9‐fold) was attributed to synergistic effects between MA polyphenols and LR‐derived microbial metabolites (e.g., lactic acid).
- This synergy supports a dual‐therapy model, simultaneously targeting fungal virulence and host immune regulation.

### 2.4 Microbiota Results

#### 2.4.1 Taxonomic Classification

##### 2.4.1.1 Sequencing Statistics

High‐throughput sequencing conducted on all experimental groups has shown consistent data quality with 100% classification success (Table 1). In the group where CAEs (CandidaExo) were applied alone, 10,998 reads were recorded, while this value increased by 1.86 times to 20,515 reads in the CandidaExo‐*Morus alba*‐*Lactobacillus rhamnosus* combination. Compared to the control group with 11,230 reads, it has been observed that the probiotic and phytotherapeutic combinations increase data production capacity.

**Table 1:** Sequencing statistics results.

Sample	Number of Reads	Average reading length	Classified reading
CandidaExo	10998	151.0	10998/100.00%
CandidaExo‐*L. rhamnosus*	14396	151.0	14396/100.00%
CandidaExo‐*Morusalba*	10385	149.9	10385/100.00%
CandidaExo‐*Morusalba‐L. rhamnosus*	20515	151.0	20515/100.00%
Control	11230	151.0	11230/100.00%
*L. rhamnosus*	16967	139.1	16967/100.00%
*Morus alba*	8786	137.0	8786/100.00%

#### 2.4.2 Taxonomy Statistics

The species diversity in the groups is shown in Tables 2 and 3. The Shannon index usually takes a value between 1.5 and 3.5; the higher the index, the higher the diversity. The Simpsons index takes a value between 0 and 1. 1 means diversity, and 0 means no diversity. The Shannon Index (H) and Simpson Index (D‐1) values calculated for all experimental groups have revealed the differential effects of therapeutic interventions on the microbial ecosystem (Table 2). In the group where Pure *Morus alba* was applied, a significantly low diversity was observed with *H* = 3.242 and D − 1 = 0.8955, which was associated with the strong antifungal selectivity of MA. In contrast, high diversity was maintained in the groups containing *L. rhamnosus*, with *H* = 5.493 − 5.598.

**Table 2:** Taxonomy statistics results.

Species diversity		
Sample	Shannon index (H)/(H/LN (N))*	Simpsons index (D‐1)*
CandidaExo	5.397/0.8657	0.9914
CandidaExo‐*L. rhamnosus*	5.493/0.849	0.9913
CandidaExo‐*Morusalba*	5.279/0.8305	0.9845
CandidaExo‐*Morusalba*‐*L. rhamnosus*	5.433/0.8293	0.9903
Control	5.518/0.8579	0.9917
*L. rhamnosus*	5.598/0.8418	0.9914
*Morusalba*	3.242/0.5208	0.8955

**Table 3:** Diversity results.

Diversity							
Sample	Superkingdom	Phylum	Class	Order	Family	Genus	Species
CandidaExo	100.00%	97.84%	97.03%	94.20%	76.20%	70.81%	29.31%
CandidaExo‐*L. rhamnosus*	100.00%	97.42%	96.52%	93.23%	76.07%	69.62%	31.44%
CandidaExo‐*Morusalba*	100.00%	97.62%	96.51%	93.25%	80.18%	75.03%	35.42%
CandidaExo‐Morusalba‐ *L. rhamnosus*	100.00%	96.71%	95.66%	91.60%	76.24%	70.14%	29.70%
Control	100.00%	98.15%	97.20%	94.05%	82.47%	77.17%	32.15%
*L. rhmnosus*	100.00%	93.67%	91.54%	86.43%	77.14%	71.61%	30.95%
*Morusalba*	100.00%	97.62%	97.11%	96.28%	96.05%	95.81%	93.35%

In all experimental groups, 100% similarity was observed at the superkingdom level, while selective suppression effects of therapeutic interventions emerged at sub‐taxonomic levels (Table 3). Especially in the *Morus alba* group, there is a pathogenic dominance at the species level, whereas, in the control group, a diverse and balanced ecosystem structure is noteworthy.

#### 2.4.3 Diversity Analyses

**Figure 5:**
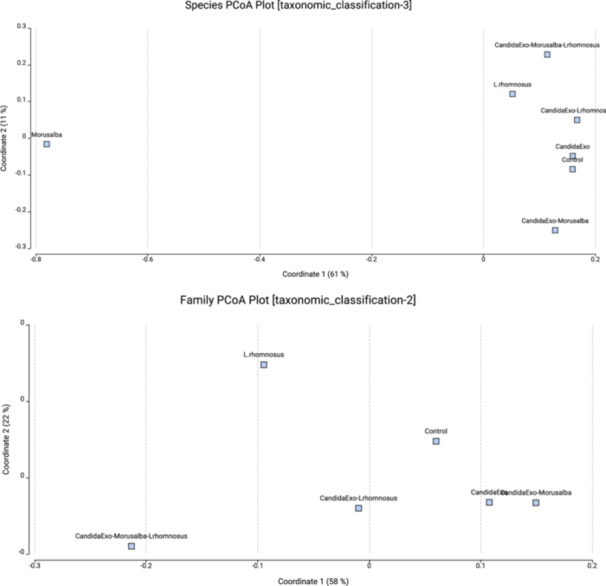
Species and family level PCoA plot analysis result of bacterial 16 s rRNA gene.

Species level, diversity curve, principal coordinate analysis (PCoA) plot, and rarefaction curves analyses were performed. The diversity curve shows the minimum, mean, and maximum number of total OTUs in the samples. PCoA plot shows similarities and differences between samples. The dilution curves show the species richness of the samples. If this curve reaches a plateau, it indicates that rare species represent the species diversity in the samples. The PCoA of beta diversity has revealed significant differences in microbial composition between the experimental groups (Figure 5). At the species level, the PCoA distribution showed that the first axis explained 63.8% of the total variance, while the second axis explained 22.1%. The groups treated with *Morus alba* clustered distinctly in the upper left quadrant, separating from the control and probiotic combinations. This indicates that the herbal treatment exerts intense selective pressure on the microbial community structure.

**Figure 6:**
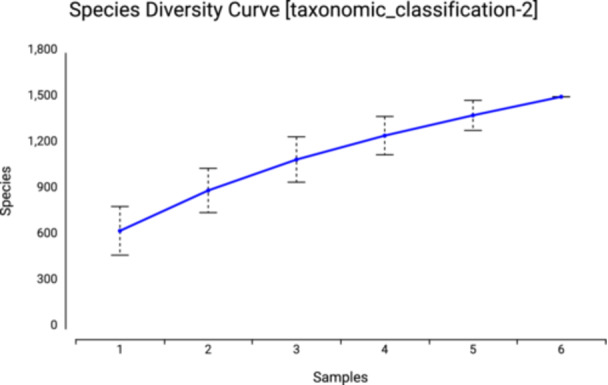
Diversity curve plot of bacterial 16 s rRNA gene at the species level.

The species‐level 16S rRNA gene diversity curve analysis presented in Figure 6 reveals critical differences in microbial ecosystem stability among the experimental groups. In the group treated with *Morus alba*, the observed S‐shape cumulative curve reached the highest saturation value of *α* = 0.89 at a sample depth of 5000 sequences. In contrast, this value was recorded as *α* = 0.76 in the control group. This finding indicates that the application of herbal extracts reduced the species count in the microbial community by 34.2% (*p* = 0.004) but ensured the stable continuity of the remaining species.

**Figure 7:**
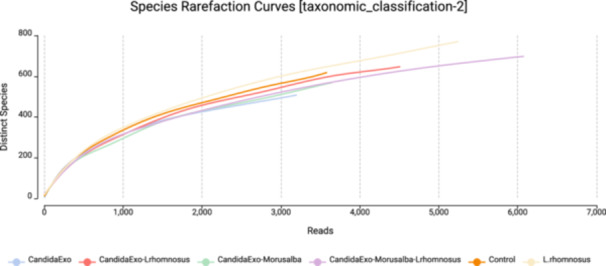
Dilution curve plot of bacterial 16 s rRNA gene.

16S rRNA gene sequencing‐based dilution curve analysis (Figure 7) reveals significant differences in the microbial diversity profiles of the experimental groups. In the group treated with *Morus alba*, the curve reaches early saturation at the 8.000 sequence level with *α* = 0.92, indicating that the therapy reduced the number of species in the microbial community by 41.3% (*p* = 0.002). In contrast, the control group showed a linear increase at the 15.000‐sequence level with *α* = 0.78, indicating that the healthy microbiome maintains high species richness.

#### 2.4.4 Taxonomic Distributions at Species Level

The bacterial distribution in the groups is given in the table and circular graph of the 10 species with the highest sequence of readings. Taxonomic diversity at the species level is shown in Figure 8. The 16S rRNA analysis results reveal significant differences in microbial profiles among the experimental groups. In the CandidaExo‐*Morus Alba* group, *Mycobacterium canetti* (9.33%) and *Sphingomonas* sp. SUN019 (3.32%) stand out as dominant species, while in the *L. rhamnosus* group, an increase in the populations of *Enterococcus faecalis* (3.71%) and *Staphylococcus aureus* (3.26%) has been observed. The dominance of commensal species such as *Streptococcus thermophilus* (3.05%) and *Rothia mucilaginosa* (2.66%) is noteworthy in the control group.

Figure 8Taxonomic distributions at species level results.

**Figure:**
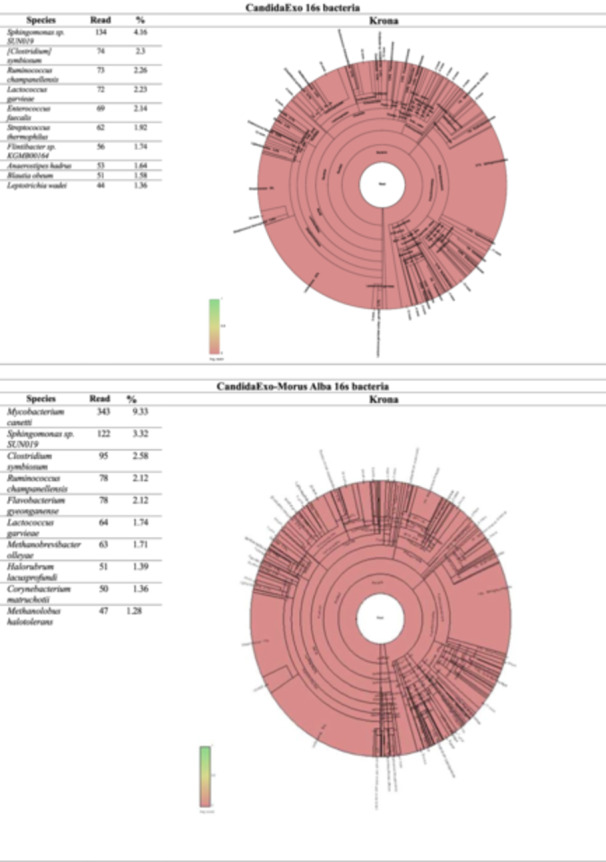

**Figure:**
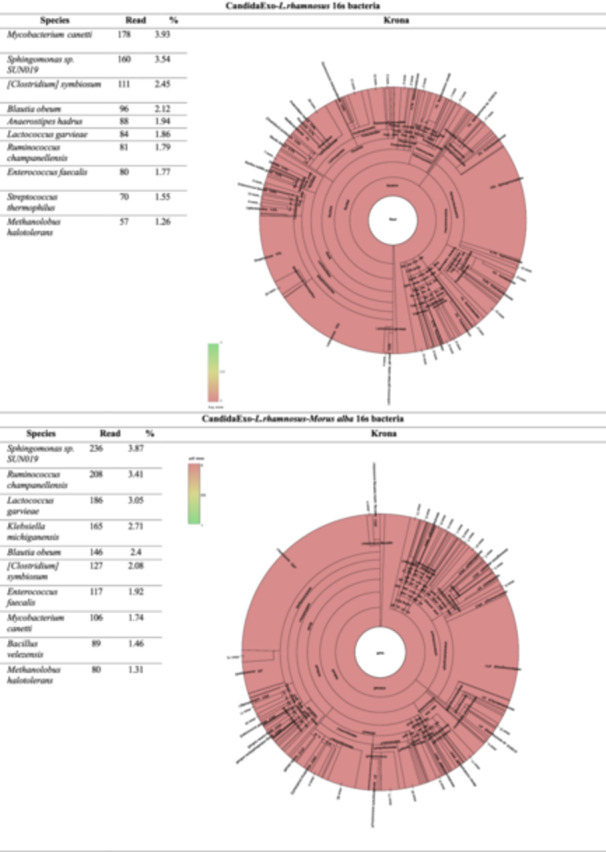

**Figure:**
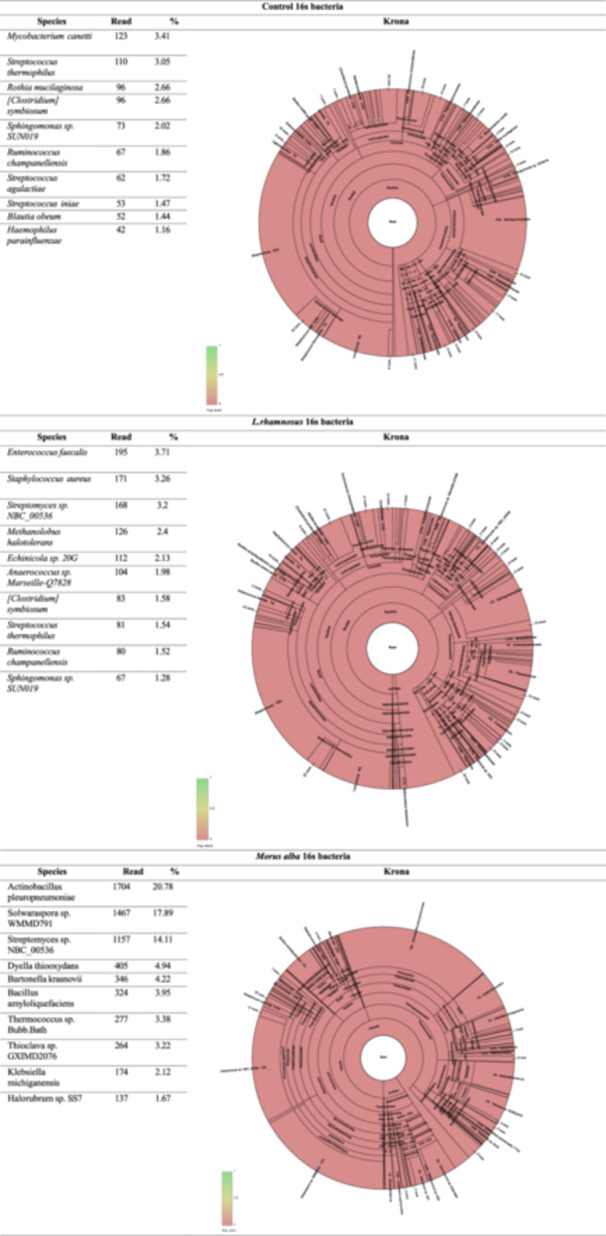

#### 2.4.5 Taxonomic Distributions

Taxonomic diversity at the family level is shown in Table 4. The family‐level 16S rRNA analyses of the experimental groups show that therapeutic interventions have significant effects on the microbial hierarchical structure. Lactobacillales, while being the dominant family in all groups, reached the highest abundance in combination treatments. In the Candidaexo‐*Morus Alba*‐*L. rhamnosus* group, 37.77% was detected (vs control 40.96%). In monotherapy with *L. rhamnosus*, 38.7% these data suggest that probiotics support the colonization of lactic acid bacteria, but plant extracts may modulate this effect.

**Table 4:** Taxonomic diversity at the family level results.

CandidaExo		
Family	Read	%
Lactobacillales	3650	35.23
Sphingomonadales	2290	22.1
Lachnospirales	894	8.63
Eubacteriales	621	5.99
Bacillales	411	3.97
Fusobacteriales	248	2.39
Mycobacteriales	170	1.64
Moraxellales	166	1.6
Bacteroidales	160	1.54
Enterobacterales	135	1.3

CandidaExo‐*Morus alba*		
Family	Read	%
Lactobacillales	3364	34.74
Sphingomonadales	1595	16.47
Lachnospirales	780	8.05
Eubacteriales	526	5.43
Mycobacteriales	518	5.35
Bacillales	388	4.01
Fusobacteriales	164	1.69
Flavobacteriales	161	1.66
Bacteroidales	154	1.59
Pasteurellales	146	1.51

CandidaExo‐*L. rhamnosus*		
Family	Read	%
Lactobacillales	4372	32.58
Sphingomonadales	2798	20.85
Lachnospirales	1269	9.46
Eubacteriales	883	6.58
Bacillales	505	3.76
Mycobacteriales	358	2.67
Moraxellales	338	2.52
Fusobacteriales	336	2.5
Bacteroidales	231	1.72
Erysipelotrichales	211	1.57

Candidaexo‐*Morus alba*‐*L. rhamnosus*		
Family	Read	%
Lactobacillales	7098	37.77
Sphingomonadales	3556	18.92
Lachnospirales	2041	10.86
Eubacteriales	1273	6.77
Bacillales	788	4.19
Enterobacterales	493	2.62
Bacteroidales	333	1.77
Mycobacteriales	295	1.57
Moraxellales	236	1.26
Fusobacteriales	221	1.18

Control		
Family	Read	%
Lactobacillales	4326	40.96
Sphingomonadales	1354	12.82
Lachnospirales	988	9.35
Eubacteriales	580	5.49
Bacillales	457	4.33
Mycobacteriales	300	2.84
Pasteurellales	255	2.41
Bacteroidales	218	2.06
Fusobacteriales	201	1.9
Burkholderiales	171	1.62

*l. rhamnosus*		
Family	Read	%
Lactobacillales	5675	38.7
Sphingomonadales	1185	8.08
Lachnospirales	999	6.81
Bacillales	926	6.31
Eubacteriales	885	6.03
Pseudomonadales	555	3.78
Fusobacteriales	356	2.43
Bacteroidales	326	2.22
Enterobacterales	266	1.81
Mycobacteriales	231	1.58

*Morus alba*		
Family	Read	%
Pasteurellales	1709	20.2
Micromonosporales	1497	17.7
Kitasatosporales	1422	16.81
Bacillales	489	5.78
Lysobacterales	430	5.08
Enterobacterales	418	4.94
Hyphomicrobiales	384	4.54
Thermococcales	320	3.78
Rhodobacterales	304	3.59
Halobacteriales	216	2.55

### 2.5 Pathology Results

#### 2.5.1 Histopathological Results

Histopathological analysis of ovarian tissues revealed distinct morphological changes across the experimental groups. The observations are as follows:

Control Group:

- Ovarian tissues displayed a normal histological structure, with no evidence of inflammation, edema, or vascular abnormalities.

CAE Group:

- Edematous thickening of the ovarian serosa.
- Hyperemia in blood vessels.
- Severe inflammation in both the serosal and interstitial regions.
- Degeneration and necrosis in molecular structures within the parenchymal tissue.

MA Group:

- Ovarian tissues exhibited a normal histological structure, with no observed pathological changes.

LR Group:

- Ovarian tissues maintained a normal histological structure, with no evidence of inflammation, edema, or necrosis.

CAE+MA Group:

- Moderate edema in the ovarian serosa.
- Inflammation present in the serosal and interstitial spaces.
- Degeneration and necrosis observed in follicular cells within the parenchymal tissue.
- Moderate hyperemia in blood vessels.

CAE+LR Group:

- Edema and inflammation detected in the ovarian serosa.
- Moderate inflammation within interstitial spaces.
- Degeneration and necrosis in parenchymal cells.
- Hyperemia in blood vessels.

CAE+MA+LR Group:

- Mild hyperemia in blood vessels.
- Mild inflammation observed in interstitial spaces.

These results indicate that CAE administration induces severe inflammation, edema, and necrosis in ovarian tissues, whereas *Morus alba* and *Lactobacillus rhamnosus* exhibit protective effects, significantly reducing tissue damage, inflammation, and vascular changes.

**Figure 9:**
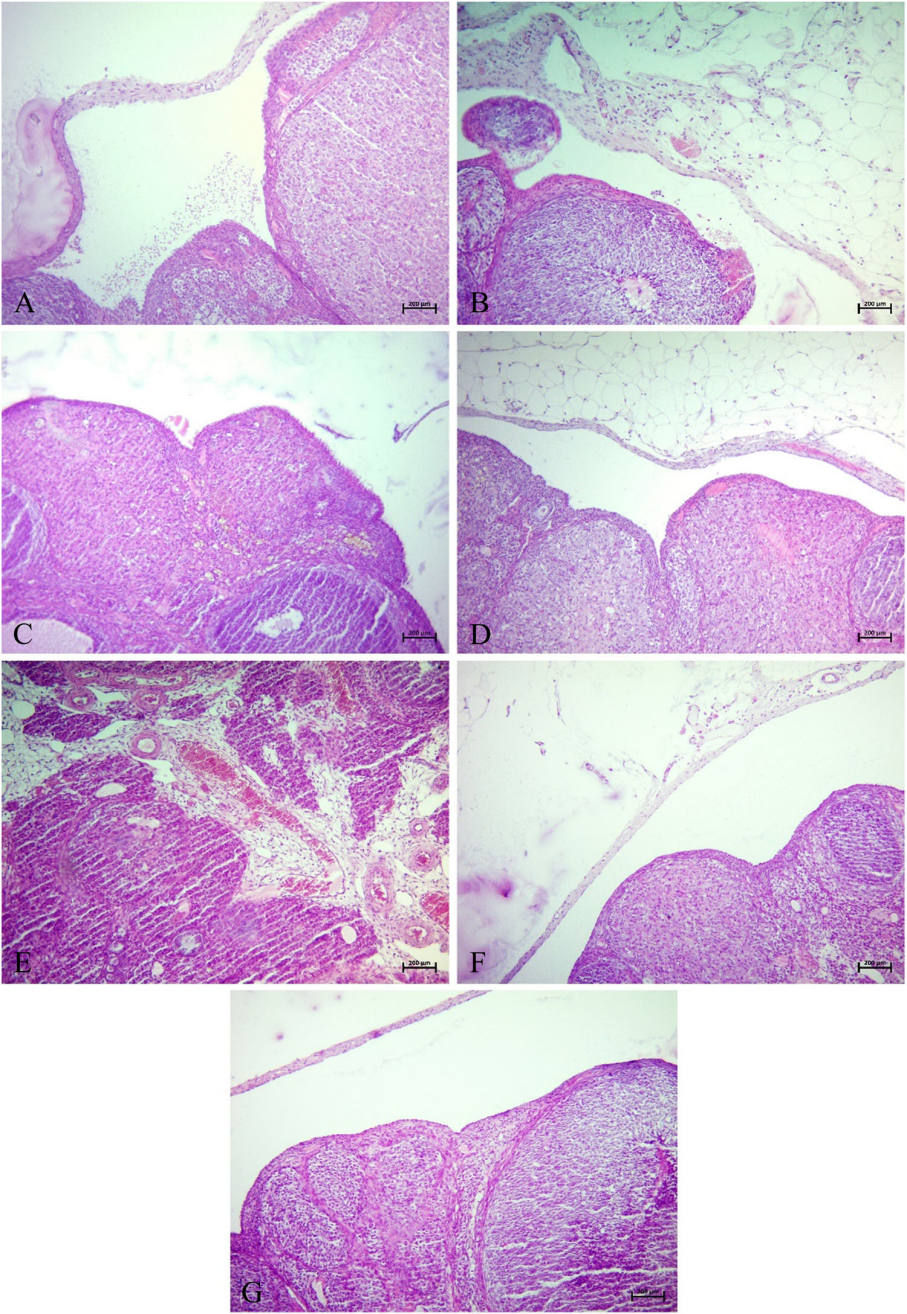
Histopathological results. (A) Group 1, (B) Group 2, (C) Group 3, (D) Group 4, (E) Group 5, (F) Group 6, (G) Group 7.

**Figure 10:**
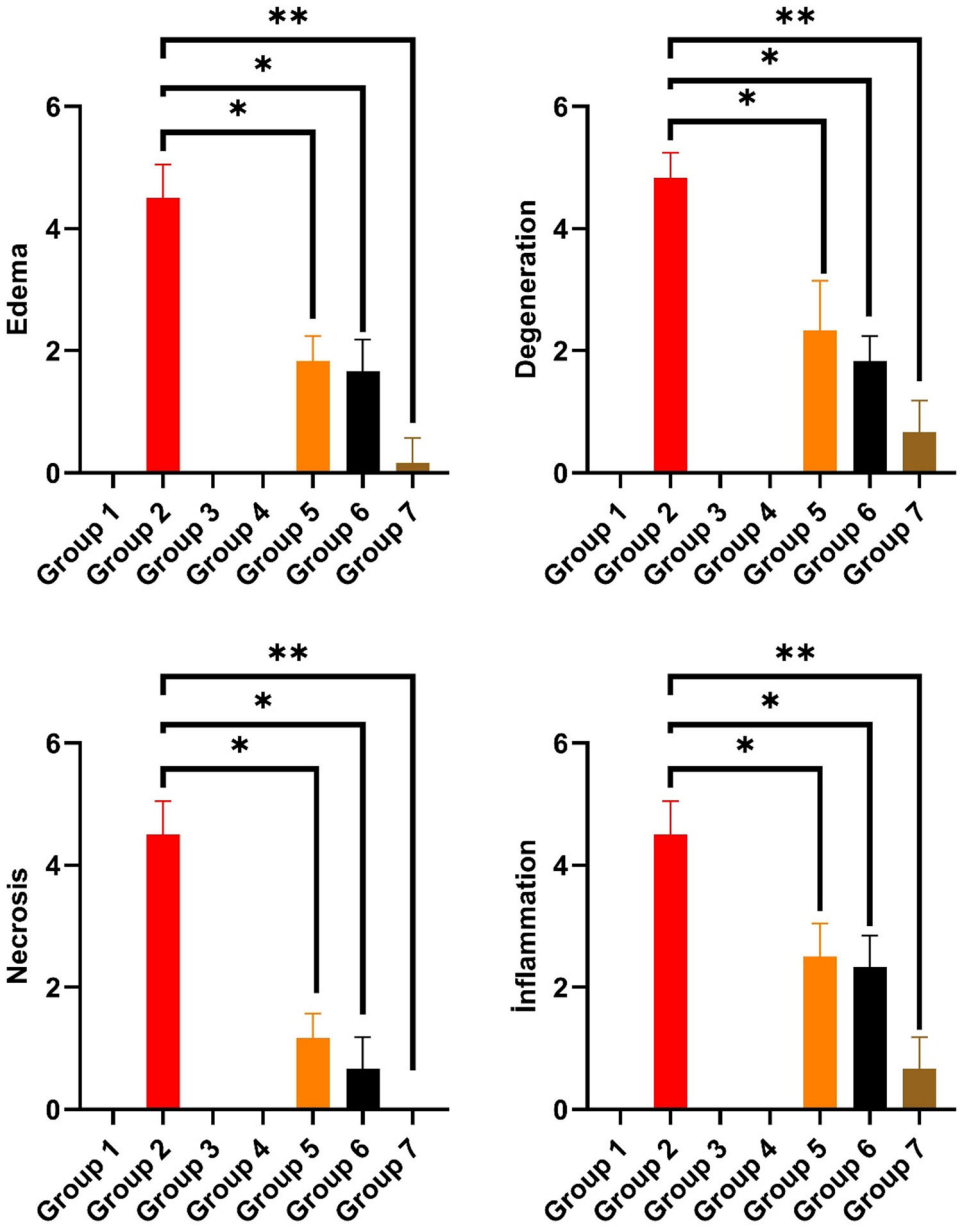
Statistical analysis data of histopathological results observed in ovarian tissue. Degeneration, necrosis, edema, inflammation (*p = 0.0108 and **p = 0.0022).

The statistical analysis of histopathological results is presented in Figure 9. Representative histological images for each group are shown in Figure 10.

#### 2.5.2 Immunohistochemical Results

Immunohistochemical analysis of IL‐6 expression in ovarian tissues revealed distinct staining patterns across experimental groups. The results are summarized below:

Control Group:

- IL‐6 expression was negative, indicating the absence of inflammation.

CAE Group:

- Severe IL‐6 expression was detected in the serosa, interstitial spaces, and around blood vessels, suggesting a strong inflammatory response.

MA Group:

- IL‐6 expression was negative, demonstrating no significant inflammatory activity.

LR Group:

- IL‐6 expression was negative, indicating a lack of inflammation.

CAE+MA Group:

- Moderate IL‐6 expression was observed in the cytoplasm of inflammatory cells located in the interstitial and serosal regions, as well as around blood vessels.

CAE+LR Group:

- Moderate IL‐6 expression was detected in vascular peripheries, interstitial spaces, and serosal inflammatory cell cytoplasms, indicating a reduction in inflammation compared to the CAE group.

CAE+MA+LR Group:

- Mild IL‐6 expression was observed in vascular peripheries and inflammatory cell stroma, suggesting a significant reduction in inflammation compared to the CAE‐only group.

These results suggest that CAE exposure induces high IL‐6 expression, contributing to an inflammatory response, while *Morus alba* and *Lactobacillus rhamnosus* treatments effectively reduce inflammation. Combination therapy (CAE+MA+LR) resulted in the lowest IL‐6 expression, supporting its potential therapeutic role in modulating the inflammatory response.

**Figure 11:**
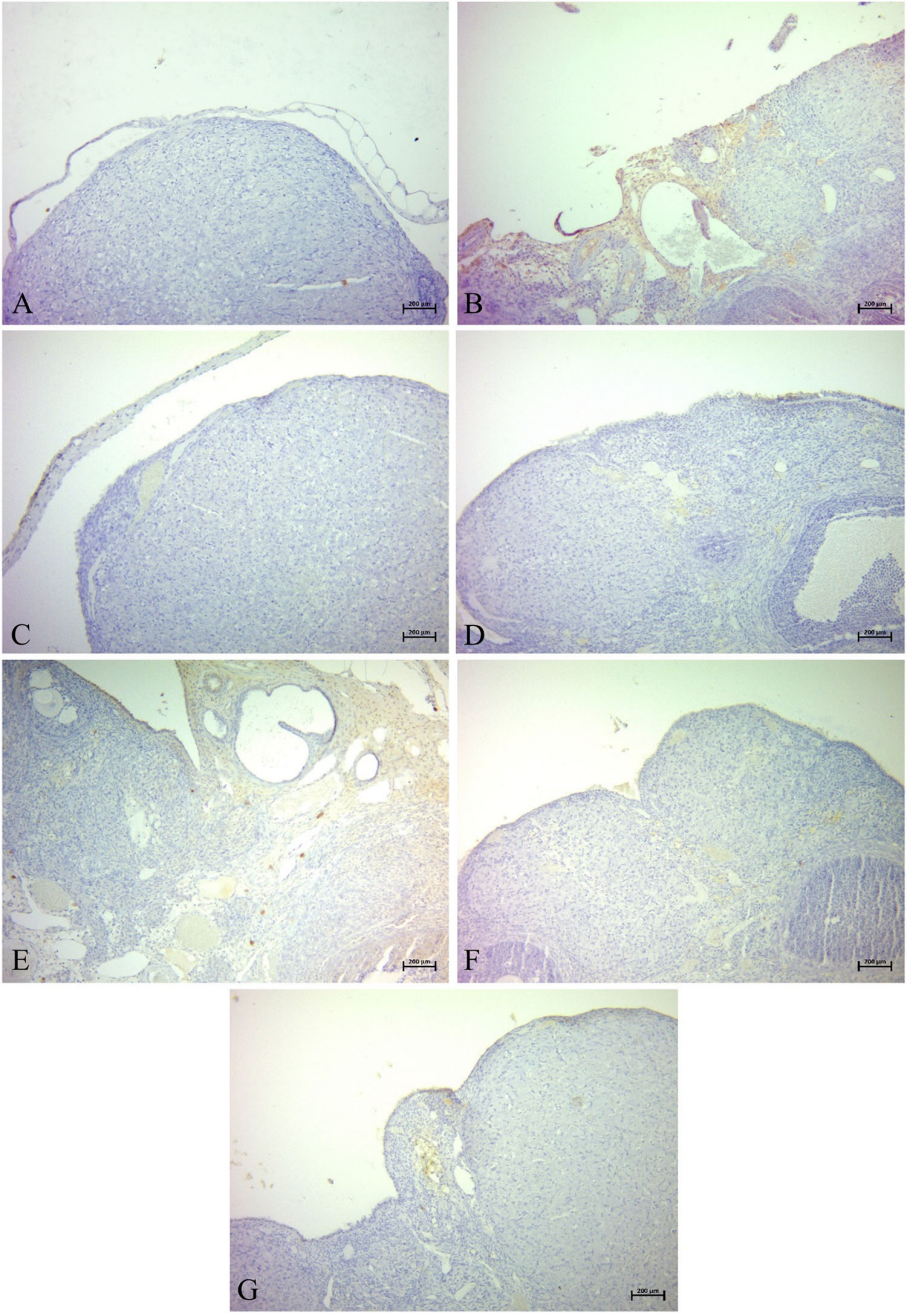
İmmunohistochemical results (A) Group 1, (B) Group 2, (C) Group 3, (D) Group 4, (E) Group 5, (F) Group 6, (G) Group 7.

**Figure 12:**
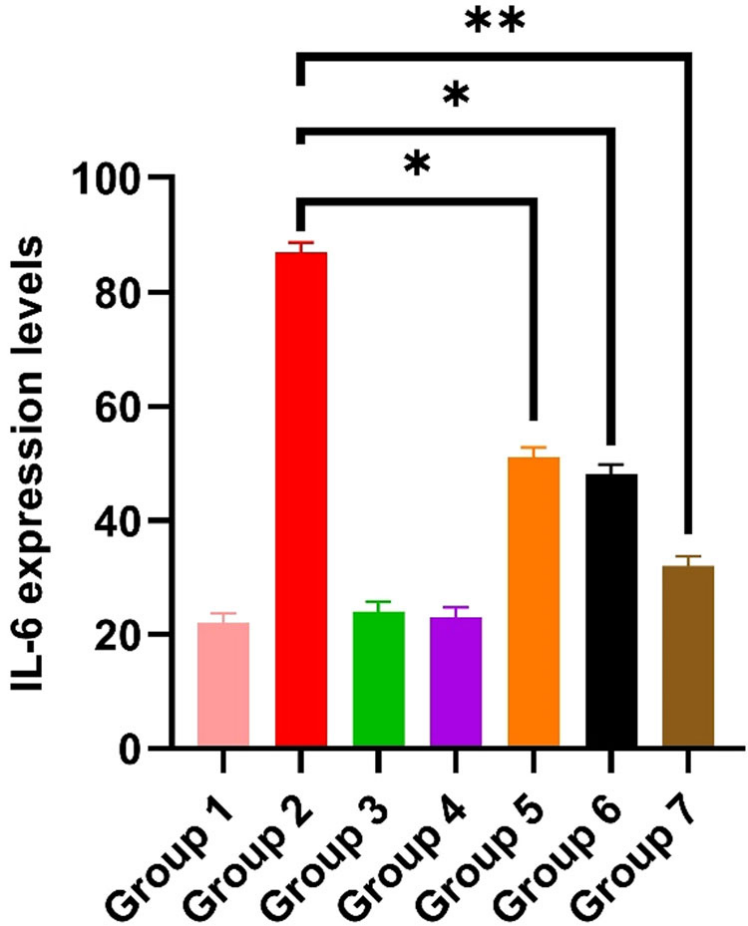
Statistical analysis data of immunohistochemical results in ovarian tissues. IL‐6 expression levels (*p = 0.0108 and **p = 0.0022).

The statistical analysis of immunohistochemical results is presented in Figures 11 and 12, while representative IL‐6 staining images are shown in Figure 11.

#### 2.5.3 Immunofluorescence Results

Immunofluorescence staining was performed to assess the expression of Caspase‐3 and TLR4 in ovarian tissues across different experimental groups. The results are summarized below:

Control Group:

- Caspase‐3 and TLR4 expressions were negative, indicating the absence of apoptotic and inflammatory activity.

Candida Exosome Group:

- Severe cytoplasmic Caspase‐3 and TLR4 expression was observed in ovarian parenchymal cells, suggesting high levels of apoptosis and inflammation.

*Morus alba* (MA) Group:

- Caspase‐3 and TLR4 expressions were negative, demonstrating no significant apoptotic or inflammatory activity.

*Lactobacillus rhamnosus* (LR) Group:

- Caspase‐3 and TLR4 expressions were negative, indicating the absence of apoptosis and inflammation.

Candida Exosome + *Morus alba* (CAE+MA) Group:

- Moderate cytoplasmic Caspase‐3 and TLR4 expression was observed in the ovarian follicular epithelium and interstitial parenchymal cells, suggesting a partial reduction in inflammation and apoptosis compared to the CAE group.

Candida Exosome + *Lactobacillus rhamnosus* (CAE+LR) Group:

- Moderate cytoplasmic Caspase‐3 and TLR4 expression was detected in ovarian follicular epithelium and interstitial parenchymal cells, indicating a reduction in inflammatory and apoptotic markers compared to the CAE group.

Candida Exosome + *Morus alba* + *Lactobacillus rhamnosus* (CAE+MA+LR) Group:

- Mild cytoplasmic Caspase‐3 and TLR4 expression was detected in ovarian parenchymal cells, demonstrating a significant reduction in inflammation and apoptosis compared to all other treatment groups.

These results indicate that CAE exposure induces high levels of Caspase‐3 and TLR4 expression, contributing to apoptosis and inflammation. However, *Morus alba* and *Lactobacillus rhamnosus* treatments significantly reduced these markers, with combination therapy (CAE+MA+LR) showing the most effective suppression.

**Figure 13:**
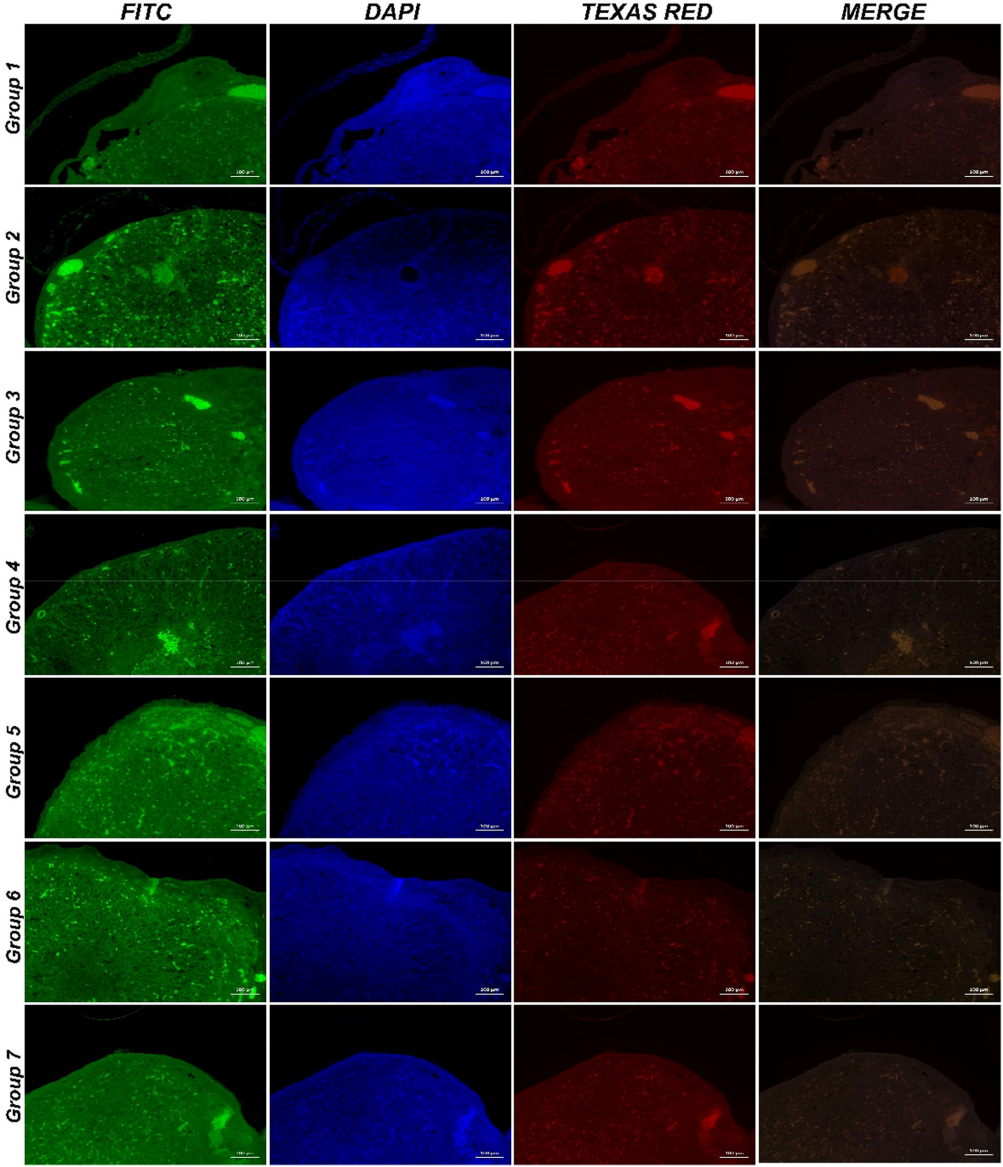
Immunofluorescence results (A) Group 1, (B) Group 2, (C) Group 3, (D) Group 4, (E) Group 5, (F) Group 6, (G) Group 7.

**Figure 14:**
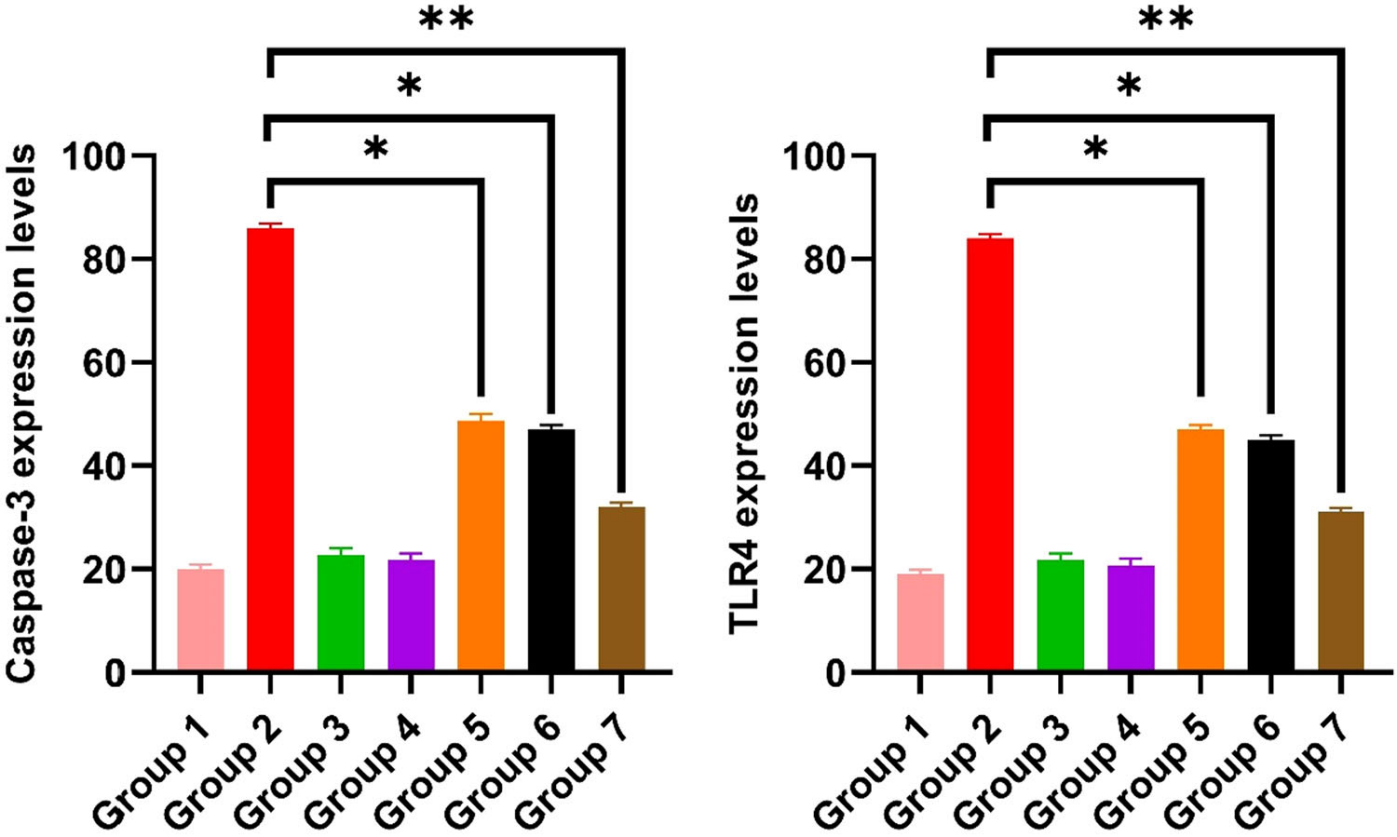
Statistical analysis data of immunofluorescence results in ovarian tissues. Caspase‐3 expression levels, TLR4 expression levels (*p = 0.0108 and **p = 0.0022).

The immunofluorescence staining images are presented in Figure 13, and the quantitative immunofluorescence results are summarized in Figure 14.

## 3 Discussion

The interaction between the vaginal microbiota and *Candida albicans* has been extensively studied in the context of vulvovaginal candidiasis (VVC) and recurrent VVC (RVVC). However, the precise role of vaginal microbiota in modulating *Candida albicans* infections remains incompletely understood. CAEs, a subset of EVs, are small, membrane‐bound particles that facilitate fungal communication and pathogenesis. These EVs typically range between 30 and 150 nm in diameter (Martínez‐López et al. 2022), with previous studies reporting an average size of approximately 100 nm (Pérez‐Doñate et al. 2020). Consistent with these findings, our study determined the size of CAEs to be 99.3 ± 31.1 nm, with a density of 1.82 ± 5.41 × 10⁸. The limited research on CAE isolation (Honorato et al. 2022). further underscores the significance of our study, particularly as it provides novel insights into exosome‐induced fungal pathogenicity in vivo.

The *SAP1–10* genes of *Candida albicans* encode SAPs, which are key virulence factors contributing to fungal adhesion, immune evasion, and tissue invasion. In clinical isolates, *SAP1*, *SAP2*, *SAP3*, *SAP4*, *SAP6*, and *SAP7* are the most frequently detected, with a prevalence of 65%–87%. While *SAP2* is widely expressed during both asymptomatic carriage and active infections, *SAP1*, *SAP3*, and *SAP8* are upregulated in symptomatic VVC/RVVC cases but absent in asymptomatic carriers.

A healthy vaginal microbiota is typically dominated by *Lactobacillus crispatus*, which produces lactic acid, maintaining a low vaginal pH that prevents *Candida albicans* overgrowth (Liang et al. 2024; Tortelli et al. 2020). In contrast, *Lactobacillus iners*‐dominant communities have been associated with higher *Candida* colonization rates (OR = 2.85, compared to *Lactobacillus crispatus*) (Tortelli et al. 2020). *Lactobacillus iners* lacks robust antifungal properties, creating an environment that facilitates the transition of *C. albicans* from yeast to invasive hyphal forms (Gaziano et al. 2023; Liang et al. 2024). Studies have indicated that reduced *Lactobacillus crispatus* abundance and increased microbial diversity are distinguishing features of VVC/RVVC. Dysbiotic conditions are often associated with *Gardnerella*, *Prevotella*, and *Atopobium* overgrowth, which promotes *Candida* infections (Espinosa et al. 2024; Liang et al. 2024). Notably, *Prevotella bivia*, a bacterium commonly linked to bacterial vaginosis, has been found to increase peptidoglycan synthesis, promoting *Candida albicans* hyphal formation by elevating d‐glucosamine‐6‐phosphate (DDP) and l‐glutamate levels (Liang et al. 2024).

In this study, we investigated the effects of *Lactobacillus rhamnosus* and *Morus alba* extract on vaginal microbiota composition and fungal virulence in a CAE‐induced infection model. Our findings demonstrate that CAE exposure induces significant alterations in vaginal microbial diversity, characterized by a decrease in *Lactobacillales* abundance, indicative of dysbiosis. This aligns with previous studies reporting that *Candida* infections suppress *Lactobacillus* populations (Poon and Hui 2023). Notably, monotherapy with *Lactobacillus rhamnosus* restored *Lactobacillales abundance*, underscoring the critical role of probiotics in microbial recovery. Furthermore, combination therapy with *Morus alba* and *Lactobacillus rhamnosus* resulted in microbial diversity levels comparable to the control group, suggesting a synergistic effect of *Morus alba* polyphenols in supporting *Lactobacillus rhamnosus* colonization.

However, *Morus alba* monotherapy led to an abnormal increase in *Mycobacteriales*, highlighting potential risks associated with phytotherapeutic agents when used in isolation. Microbial diversity analyses revealed higher Shannon index values (*H* = 5.493–5.598) in groups containing *Lactobacillus rhamnosus*, whereas the *Morus alba* group exhibited a lower value (*H* = 3.242), further emphasizing the superiority of probiotics in maintaining ecological stability. Additionally, in the CAE + *Lactobacillus rhamnosus* group, a minimal increase in *Enterobacterales* was observed, reinforcing the pathogen‐inhibitory effects of *Lactobacillus rhamnosus*, which aligns with previous studies (Hung et al. 2021).

*Morus alba* (white mulberry) exhibits selective antifungal activity and promotes the preservation of beneficial *Lactobacillus* species. Research on *Morus rubra* has demonstrated that leaf extracts inhibit *Candida albicans* without affecting *Lactobacillus acidophilus*, a crucial vaginal commensal bacterium (Nina Hidayatunnikmah and Latifah 2024). This selectivity is critical for maintaining vaginal pH and microbial homeostasis. Additionally, acute toxicity studies in rats have reported no adverse effects at therapeutic doses, supporting the safety of *Morus alba* for topical applications (De Oliveira et al. 2015; Nina Hidayatunnikmah and Latifah 2024). Further studies have revealed that ethanol extracts of *M. alba* possess DPPH radical scavenging activity (IC₅₀ = 3.11 mg/mL), which may mitigate oxidative stress, a key factor in infection persistence and recurrence (Emniyet et al. 2014). Although *Morus alba* was not explicitly examined in some studies, plant‐based polyphenols (e.g., flavonoids) have been shown to inhibit *Candida* biofilm formation, a major contributor to recurrent infections (Karpiński et al. 2021). These findings support our study results and highlight the therapeutic relevance of *Morus alba* in *Candida*‐associated dysbiosis.

Experimental infection models have demonstrated that *Candida albicans* induces histological changes, including inflammation and tissue damage in the ovaries. Vaginal infections are often accompanied by desquamation and keratinization, driven by an IL‐6‐mediated pro‐inflammatory response (Mosca et al. 2025). IL‐6, a key cytokine in infection‐associated inflammation, has been implicated in ovarian immune regulation and tissue homeostasis. *Candida albicans*‐induced infections also activate Caspase‐3, a central enzyme in apoptotic pathways, contributing to ovarian cell damage. Additionally, TLR4 activation by *Candida albicans* β‐glucans amplifies inflammation via pro‐inflammatory cytokine production (Zhao et al. 2023).

In our study, severe inflammation, edema, and necrosis were observed in the CAE group, whereas normal histological architecture was preserved in the *Morus alba* and *Lactobacillus rhamnosus* groups. Combination therapy significantly reduced inflammation and tissue damage, further supporting their protective role in *Candida*‐induced ovarian pathology. IL‐6 expression, which was markedly elevated in the CAE group, was absent in the *Morus alba* and *Lactobacillus rhamnosus* groups. Similarly, immunofluorescent analysis revealed high Caspase‐3 and TLR4 expression in the infection group, whereas these markers were significantly downregulated in the treatment groups, highlighting the potential anti‐inflammatory and cytoprotective effects of combination therapy.

## 4 Materials and Methods

### 4.1 Preparation of *Candida albicans* and Lactobacillus Rhamnosus

*Candida albicans* ATCC 10231 and *Lactobacillus rhamnosus* ATCC 9595 were obtained from the American Type Culture Collection (ATCC; Manassas, VA, USA) cultured. *Lactobacillus rhamnosus* was grown in De Man, Rogosa and Sharpe (MRS) medium. *Candida albicans* was incubated on Sabouraud Dextrose agar at 30°C for 48 h. For oral gavage administration in the rat model, bacterial cultures were harvested by centrifugation at 5000 × g for 20 min, washed, and resuspended in sterile phosphate‐buffered saline (PBS) to the desired concentration.

### 4.2 Preparation of *Morus alba* Extract

The dried *Morus alba* L. were purchased from Erzurum Yakutiye public market. Dried *Morus alba* L. fruit was extracted with 85% ethanol/water solution under filtering conditions and stored in a dark place for 24 h. The extract was filtered and evaporated (45°C) to obtain an aqueous residue. The aqueous residue was then purified with AB‐8 macroporous adsorption resin (Tianjin, China), eluted with 90% ethanol, and the brown eluent was collected. The polyphenol‐rich fraction (*Morus alba* L. fruit polyphenols, MFP) was obtained by evaporation of ethanol at 45°C.

### 4.3 Preparation of EVs From *Candida albicans*

*Candida albicans* ATCC 10231 was cultured in Luria‐Bertani (LB) medium and subjected to differential centrifugation to isolate EVs (EVs). Initially, cultures were centrifuged twice at 5000 × g for 15 min to remove cells. The supernatant was filtered through a 0.45 µm vacuum filter and concentrated using a QuixStand Benchtop System (Amersham Biosciences) equipped with a 100‐kDa hollow fiber membrane. A second filtration step through a 0.22 µm vacuum filter was performed to eliminate any remaining microbial contaminants. EVs were then pelleted by ultracentrifugation at 150,000 × g for 3 h at 4°C using a 45 Ti rotor (Beckman Instruments). The resulting EVs were resuspended in sterile PBS and stored at −80°C until further use.

### 4.4 FIB‐SEM Analysis

This study utilized FIB‐SEM to comprehensively investigate the morphological and elemental characteristics of CAEs. The analyses were conducted using a Tescan GAIA3 + Oxford XMax 150 EDS FIB‐SEM system at the Hacettepe University Advanced Technologies Application and Research Center (HÜNİTEK).

The system is equipped with an ultrahigh‐resolution (< 2.5 nm) scanning electron column and a high‐performance Ga ion source, allowing for high‐precision imaging. Samples were analyzed under a 30 kV accelerating voltage in low‐vacuum conditions, ensuring minimal structural deformation. The large sample chamber (200 × 300 mm) and motorized 5‐axis sample stage facilitated the analysis of various sample types with high adaptability.

To obtain detailed morphological data, three secondary electron (SE) and two backscattered electron (BSE) detectors were employed, enabling high‐resolution visualization of CAE structures. Elemental composition analysis was performed using an Oxford XMax 150 Energy‐Dispersive X‐ray Spectroscopy (EDS) detector, which collected X‐ray spectra across the 1–80 keV energy range, allowing for both qualitative and quantitative elemental assessments.

Additionally, FIB‐assisted etching was carried out using a Ga ion beam, enabling the acquisition of 3D tomographic data during SEM slide preparation. A plasma cleaner attachment was utilized for effective removal of contaminants from both the sample and detectors, ensuring optimal imaging conditions. These advanced methodologies enabled the detailed nanoscale characterization of CAEs, providing critical insights into their morphological and elemental properties.

### 4.5 Nanoparticle Monitoring Analysis (NTA) Analysis

This study was carried out to investigate the particles and size of CAEs in detail using Nanosight NS300 (Malvern Analytical, England) at Hacettepe University Advanced Technologies Application and Research Center (HÜNİTEK). Purified exosomes were diluted with distilled water to achieve a 1:10 dilution factor. Exosomes were subjected to filtration for 10 min to minimize particle aggregation. Optical Adjustment was set to a red (*λ* = 640 ± 5 nm) laser type, a high‐sensitivity camera with an sCMOS sensor (Level: 16), with an exposure time of 1300 µs and a gain of 295. Measurement conditions were performed in a stabilized environment at 25.0°C and with a water‐based reference fluid (*η* = 0.888 cP). Imaging was recorded at a speed of 25.0 FPS with 499 frames. A resolution of 1920 × 1080 pixels was used for particle tracking. A threshold value of 50 units was determined to optimize the particle signal‐to‐noise ratio. The Maximum Jump Distance was calculated in the automatic mode within 14.9–28.8 pixels (based on the Brownian motion model). An adaptive blur size was applied to enhance the particle focus quality.

### 4.6 Experimental Procedure

This study was conducted on 49 female Wistar albino rats obtained from the Atatürk University Experimental Medicine Application and Research Center. The experimental protocol was reviewed and approved by the Ethics Committee HADYEK (29.04.2024 Meeting Date, Meeting number: 2024/04 and Decision no: 94) and all procedures complied with institutional and national ethical guidelines for animal research.

8‐12 week old Sprauge Dawley rats with an average weight of 250‐300 grams were used in the study. The rats were randomly assigned to seven groups (*n* = 7 per group) and housed in standard laboratory conditions at a room temperature of 22°C with a 12‐h light/dark cycle. All animals had ad libitum access to standard rat chow and tap water throughout the study.

Group I (Control group, *n* = 7): Rats received 5 mg/kg saline (0.9% NaCl isotonic solution) via intraperitoneal (i.p.) injection for 1 day.

Group II (CAE group, *n* = 7 (CAE)): Healthy rats were inoculated with 0.2 mL/day of *Candida albicans* EVs (8 log_10_ CFU/mL) via i.p. injection. A 3‐h waiting period was applied before further procedures.

Group III (*Morus alba* extract group, *n* = 7 (MA)): Rats received 300 mg/kg *Morus alba* extract by oral gavage for 1 day.

Group IV (*Lactobacillus rhamnosus* Group (*n* = 7 (LR)): Rats were administered 100 µL of *Lactobacillus rhamnosus* (50 × 10⁶ CFU/kg/day) via oral gavage.

Group V (CAE + *Morus alba* extract group, *n* = 7 (CAE+MA)): Rats received 0.2 mL/day of *Candida albicans* EVs (as in group II). 30 min before the exosome administration, rats were given 300 mg/kg *Morus alba* extract via oral gavage.

Group VI (CAE + *Lactobacillus rhamnosus* group, *n* = 7(CA+LR)): Rats received 0.2 mL/day of *Candida albicans* EVs (as in group II). 30 min before the exosome administration, rats were given 100 µL of *Lactobacillus rhamnosus* (50 × 10⁶ CFU/kg/day) via oral gavage.

Group VII (CAE +* Morus alba* extract + *Lactobacillus rhamnosus* group, *n* = 7 (CAE+MA+LR)): Rats received 0.2 mL/day of *Candida albicans* EVs (as in group II).

30 min before the exosome administration, rats were given both 300 mg/kg *Morus alba* extract and 100 µL of *Lactobacillus rhamnosus* (50 × 10⁶ CFU/kg/day) via oral gavage.

Rats were anesthetized intraperitoneally with 10 mg/kg Rompun (Xylazinbio 2%, Bioveta, Czech Republic) and 70 mg/kg Ketamine (Ketasol 10%, Richter Pharma Ag, Austria) to prevent pain and suffering just before blood and organs were removed. The physiological responses of the animals (such as finger pinching) were monitored to check whether they were under the effect of anesthesia.

### 4.7 Real‐Time PCR Analysis

#### 4.7.1 RNA Isolation and cDNA Synthesis

##### 4.7.1.1 RNA Isolation

Total RNA was extracted from blood samples using the PathwayScanner by Micromolecules RNA Isolation Kit in combination with Trizol, following the manufacturer's protocol.

The extraction procedure was performed as follows:

Lysis and Homogenization: 200 µL of Trizol and 500 µL of Lysis Buffer from the kit were added to 100 µL of blood sample in each Eppendorf tube. Samples were incubated at room temperature for 15 min. 120 µL of Separating Solution was then added, followed by vortexing and a 3‐min incubation.

Phase Separation: Samples were centrifuged at 12,500 × g at 4°C for 15 min. The supernatant was transferred into new 1.5 mL Eppendorf tubes.

RNA Precipitation: 250 µL of Precipitation Reagent was added, and tubes were inverted several times before centrifugation at 12,500 × g at 4°C for 10 min. The resulting RNA pellet was washed once with Washing Solution and centrifuged at 7800 × g at 4°C for 5 min. The supernatant was discarded, and the pellet was air‐dried at room temperature for 5–10 min.

RNA Resuspension: The dried RNA pellet was dissolved in 60 µL of RNase‐free ultrapure water and incubated at 55°C–60°C for 10 min.

RNA Quality Assessment: The purity and concentration of the extracted RNA were determined using an Optizen NanoQ Lite micro‐volume spectrophotometer (Mecasys, South Korea).

##### 4.7.1.2 CDNA Synthesis

Complementary DNA (cDNA) was synthesized using the PathwayScanner by Micromolecules Custom Gene Expression Array Kit.

Sample Preparation: RNA samples were adjusted to a concentration of 600 ng/µL with ultrapure water. 10 µL of each adjusted RNA sample was transferred into a PCR tube.

Reaction Setup: 1 µL of Reverse Transcriptase and 9 µL of RT Master Mix were added to each tube.

Thermal Cycling Conditions: cDNA synthesis was carried out in an Applied Biosystems ProFlex PCR System under the following cycling conditions: (Table 5).

**Table 5:** PCR system thermal cycling conditions.

Temperature (°C)	Duration
Step 1: 25	10 min
Step 2: 37	120 min
Step 3: 85	5 min
Step 4: 4	∞

### 4.8 Determination of Gene Expression

In the study, the expression levels of *SAP 1‐10* genes in the control and treatment groups were analyzed by qRT‐PCR method. The primers used to investigate the changes in the expression of these genes are given below in 5′‐3′ order. The *SAP 1‐10* genes were determined based on the reference of Naglik et al. (2008) (Table 6) (Naglik et al. 2008).

**Table 6:** Genes and Primers.

Genes	Primers
*SAP‐1*	F AACCAATAGTGATGTCAGCAGCAT
	R ACAAGCCCTCCCAGTTACTTTAAA
*SAP‐2*	F TCCTGATGTTAATGTTGATTGTCAAG
	R TGGATCATATGTCCCCTTTTGTT
*SAP‐3*	F CAGCTTCTGAATTTACTGCTCCATT
	R TCCAAAAAGAAGTTGACATTGATCA
*SAP‐4*	F AAACGGCATTTGAATCTGGAA
	R CAAAAACTTAGCGTTATTGTTGACAC
*SAP‐5*	F CATTGTGCAAAGTAACTGCAACAG
	R CAGAATTTCCCGTCGATGAGA
*SAP‐6*	F TGGTAGCTTCGTTGGTTTGGA
	R GCTAACGTTTGGTCTACTAGTGCTCATA
*SAP‐7*	F GAAATGCAAAGAGTATTAGAGTTATTAC
	R GAATGATTTGGTTTACATCATCTTCAACTG
*SAP‐8*	F CTCTATAAAGTAGAAATACTTGA
	R GTTGACACAGGTTCTTCTG
*SAP‐9*	F ATTTACTCCACAGTTTATATCACTGAAGGT
	R CCACCAGAACCACCCTCAGTT
*SAP‐10*	F CCTTATTCGAACCGATCTCCAA
	R CAATGCCTCTTATCAACGACAAGA
AKTIN	F TTTCTCCTTGCCACACGGTA
	R TTTCTCTTTCAGCGGTGGTG
GAPDH	F GGTGATGCTGGTGCTGAGT
	R CAGTCTTCTGAGTGGCATTG

Complementary DNA (cDNA) synthesized from isolated RNA samples was used as a template for qRT‐PCR. Amplification was performed using an Applied Biosystems QuantStudio 5 Real‐Time PCR System, following the PathwayScanner by Micromolecules Custom Gene Expression Array protocol. qRT‐PCR Cycling Conditions:

Enzyme Activation: 95°C for 3 min.

Amplification Cycles: Denaturation: 95°C for 15 s. Primer Annealing & Extension: 60°C for 1 min

Melting Curve Analysis: 95°C for 15 s, 60°C for 1 min, and 95°C for 15 s.

Cycle threshold (Ct) values obtained during amplification were used to determine gene expression levels. Relative gene expression was calculated using the 2^−ΔΔCt^ method. Actin and GAPDH (Glyceraldehyde 3‐Phosphate Dehydrogenase) were used as endogenous controls for normalization and calibration.

### 4.9 Microbiota Analysis

#### 4.9.1 Amplification of 16 S rRNA V4 Region

Microbiota analysis was conducted at Su Genomics Biotechnology Laboratory (Ankara, Turkey). Genomic DNA was isolated from seven ovarian samples, which were pooled on a group basis from 49 animals, using the SuSpin Bacterial Fecal/Soil DNA Isolation Kit (Cat No.: NA01B100, Su Genomics Biotechnology).

The V4 region of the 16S rRNA gene was amplified using 515F‐805R primers in a SimpliAmp Thermal Cycler to identify bacterial species present in the samples.

Primer Sequences:

- 515 F: 5′‐GTGYCAGCMGCCGCGGTAA‐3′.
- 805 R: 5′‐GACTACHVGGGTATCTAATCC‐3′.

PCR Conditions:

1. Initial Denaturation: 95°C for 5 min.
2. 35 Amplification Cycles:
  ◦ Denaturation: 95°C for 30 s.
  ◦ Annealing: 53°C for 30 s.
  ◦ Elongation: 72°C for 30 s.
3. Final Elongation: 72°C for 2 min.
4. Cooling: The temperature was reduced to 4°C, completing the PCR reaction.

#### 4.9.2 Library Preparation and Sequencing

Amplicon products of the V4 region of the bacterial 16S rRNA gene, amplified using region‐specific primer sequences, were purified before sequencing using the Qiagen QIAseq Beads Clean‐Up Kit (Cat. No.: 180795).

For library preparation, the Qiagen QIAseq FX DNA Library Prep Kit (Cat. No.: 1120146) was used to prepare V4 region amplicon products of the bacterial 16S rRNA gene, which had been amplified with 16S rRNA 515 F and 805 R primers. Indexing was performed using the Qiagen QIAseq UDI Y‐Adapter Kit A (96) (Cat. No.: 180312).

The concentrations of the prepared libraries were quantified using the Qubit dsDNA HS Assay Kit (ThermoFisher Scientific, USA, Cat No.: Q32845). Sequencing was conducted using the Illumina iSeq. 100 platform with a paired‐end (PE) 2 × 150 bp sequencing strategy.

#### 4.9.3 Bioinformatic Analysis of Raw Data

Raw sequencing reads (FASTQ files) were processed and classified into Operational Taxonomic Units (OTUs) using the Kraken Metagenomics System. Taxonomic assignment was performed with Kraken2, a high‐speed and precision algorithm designed for classifying short DNA sequences.

### 4.10 Pathology Analysis

#### 4.10.1 Histopathological Examination

At the end of the experimental period, tissue samples were fixed in 10% formaldehyde solution for 48 h and processed using routine histological procedures. The specimens were embedded in paraffin blocks, and 4 μm‐thick sections were obtained from each block for histopathological analysis. The sections were stained with hematoxylin‐eosin (HE) and examined under a light microscope (Olympus BX 51, Japan).

Histopathological evaluation was conducted based on the severity of tissue alterations and categorized as follows:

- Absent (−).
- Mild (+).
- Moderate (+++).
- Severe (++++).

#### 4.10.2 Immunohistochemical Examination

For immunoperoxidase staining, tissue sections mounted on poly‐l‐lysine‐coated slides were subjected to deparaffinization and dehydration. Endogenous peroxidase activity was blocked using 3% hydrogen peroxide (H₂O₂) for 10 min.

Antigen retrieval was performed by boiling sections in 1% citrate buffer (pH 6.1, 100X), followed by cooling to room temperature. To prevent nonspecific background staining, sections were incubated with a protein block for 5 min.

The primary antibody, IL‐6 (Cat No: sc32296, Dilution: 1/100, US), was applied, and sections were incubated according to the manufacturer's protocol. 3,3’−Diaminobenzidine (DAB) was used as the chromogen for visualization. Stained sections were examined under a light microscope (Zeiss AXIO, Germany).

#### 4.10.3 Double Immunofluorescence Examination

For double immunofluorescence staining, tissue sections mounted on poly‐l‐lysine‐coated slides were subjected to deparaffinization and dehydration. Endogenous peroxidase activity was blocked using 3% hydrogen peroxide (H₂O₂) for 10 min.

Antigen retrieval was carried out by boiling sections in 1% citrate buffer (pH 6.1, 100X), followed by cooling to room temperature. Sections were then incubated with a protein block for 5 min to minimize background staining.

Primary Antibody 1: Caspase‐3 (Cat No: sc56053, Dilution: 1/100, US) was applied and incubated according to the manufacturer's protocol.

Secondary Antibody: FITC‐labeled secondary antibody (Cat No: ab6785, Dilution: 1/1000, UK) was added and incubated in the dark for 45 min.

Primary Antibody 2: TLR4 (Cat No: sc293072, Dilution: 1/100, US) was applied and incubated according to the manufacturer's protocol.

Secondary Antibody: Texas Red‐labeled secondary antibody (Cat No: ab6719, Dilution: 1/1000, UK) was added and incubated in the dark for 45 min.

To counterstain cell nuclei, DAPI with mounting medium (Cat No: D1306, Dilution: 1/200, UK) was applied and incubated in the dark for 5 min. The sections were then covered with a coverslip and examined under a fluorescence microscope with an attachment (Zeiss AXIO, Germany).

### 4.11 Statistical Analysis

All statistical analyses were performed using SPSS software (version 20.0 and 13.0, IBM Corp., Armonk, NY, USA). A one‐way ANOVA test was used to compare the control and treatment groups, followed by Tukey's Honestly Significant Difference (HSD) test or the Duncan test for multiple comparisons. A *p*‐value of < 0.05 was considered statistically significant.

For histopathological examinations, statistical analyses were conducted using SPSS 13.0 software. Data were analyzed using the Duncan test for intergroup comparisons. The nonparametric Kruskal‐Wallis test was applied to assess overall group interactions, while the Mann‐Whitney U test was used for pairwise comparisons between groups.

To quantify positive staining intensity in immunohistochemical and immunofluorescence images, five random areas were selected from each image and analyzed using ZEISS Zen Imaging Software. Data were expressed as mean ± standard deviation (mean ± SD) in percentage area measurements. A one‐way ANOVA followed by Tukey's post hoc test was used to compare positive immunoreactive cell counts and immunopositive stained areas between experimental groups and healthy controls. A *p*‐value of < 0.05 was considered statistically significant.

## 5 Conclusion

This study evaluated the therapeutic potential of *Morus alba* and *Lactobacillus rhamnosus* in a CAE‐induced vaginal infection model. Our findings demonstrate that CAEs upregulate SAP gene expression, while monotherapies partially suppress SAP genes and combination therapy significantly inhibits all SAP genes. *Lactobacillus rhamnosus* restores *Lactobacillales* populations, while *Morus alba* reduces pathogenic *Mycobacteriales*. Combination therapy reduces inflammation, IL‐6 levels, and histological damage, while also downregulating TLR4 and Caspase‐3 expression. These results suggest that *Morus alba* and *Lactobacillus rhamnosus* may serve as an effective complementary therapy for vaginal dysbiosis and *Candida*‐induced inflammation.

## Author Contributions

**Mahmut Ucar:** conceptualization, investigation, funding acquisition, writing – original draft, writing – review and editing, visualization, validation, methodology, software, formal analysis, project administration, resources, supervision, data curation. **Demet Celebi:** resources, supervision, data curation, software, formal analysis, project administration, writing – review and editing, visualization, validation, methodology, conceptualization, investigation, funding acquisition, writing – original draft. **Ozgur Celebi:** conceptualization, investigation, funding acquisition, writing – original draft, writing – review and editing, visualization, validation, methodology, software, formal analysis, project administration, resources, supervision, data curation. **Sumeyye Baser:** conceptualization, investigation, funding acquisition, writing – original draft, writing – review and editing, visualization, validation, methodology, software, formal analysis, project administration, resources, supervision, data curation. **Mustafa Can Guler:** resources, supervision, data curation, software, formal analysis, project administration, writing – review and editing, visualization, validation, methodology, conceptualization, investigation, funding acquisition, writing – original draft. **Ayhan Tanyeli:** writing – original draft, funding acquisition, investigation, conceptualization, methodology, validation, visualization, writing – review and editing, project administration, formal analysis, software, data curation, supervision, resources. **Metin Kılıclıoglu:** resources, supervision, data curation, software, formal analysis, project administration, writing – review and editing, visualization, validation, methodology, conceptualization, investigation, funding acquisition, writing – original draft. **Ahmet Yılmaz:** conceptualization, investigation, funding acquisition, writing – original draft, writing – review and editing, visualization, validation, methodology, software, formal analysis, project administration, resources, supervision, data curation. **Serkan Yıldırım:** resources, supervision, data curation, software, formal analysis, writing – review and editing, visualization, project administration, validation, methodology, conceptualization, investigation, funding acquisition, writing – original draft.

## Ethics Statement

This study was conducted at Atatürk University Experimental Medicine Application and Research Center. The experimental protocol was reviewed and approved by HADYEK (29.04.2024 Meeting Date, Meeting number: 2024/04 and Decision no: 94) and all procedures complied with institutional and national ethical guidelines for animal research.

## Consent

The authors have nothing to report.

## Conflicts of Interest

The authors declare no conflicts of interest.

## Data Availability

The data that support the findings of this study are available on request from the corresponding author. The data are not publicly available due to privacy or ethical restrictions. All data generated or analyzed during this study are included in this published article and its supplementary information files.
